# Looking at My Own Face: Visual Processing Strategies in Self–Other Face Recognition

**DOI:** 10.3389/fpsyg.2018.00121

**Published:** 2018-02-13

**Authors:** Anya Chakraborty, Bhismadev Chakrabarti

**Affiliations:** Centre for Integrative Neuroscience and Neurodynamics, School of Psychology and Clinical Language Sciences, University of Reading, Reading, United Kingdom

**Keywords:** physical self-representation, self-face, autism spectrum disorder, visual processing, eye-tracking, psychophysics

## Abstract

We live in an age of ‘selfies.’ Yet, how we look at our own faces has seldom been systematically investigated. In this study we test if the visual processing of the highly familiar self-face is different from other faces, using psychophysics and eye-tracking. This paradigm also enabled us to test the association between the psychophysical properties of self-face representation and visual processing strategies involved in self-face recognition. Thirty-three adults performed a self-face recognition task from a series of self-other face morphs with simultaneous eye-tracking. Participants were found to look longer at the lower part of the face for self-face compared to other-face. Participants with a more distinct self-face representation, as indexed by a steeper slope of the psychometric response curve for self-face recognition, were found to look longer at upper part of the faces identified as ‘self’ vs. those identified as ‘other’. This result indicates that self-face representation can influence where we look when we process our own vs. others’ faces. We also investigated the association of autism-related traits with self-face processing metrics since autism has previously been associated with atypical self-processing. The study did not find any self-face specific association with autistic traits, suggesting that autism-related features may be related to self-processing in a domain specific manner.

## Introduction

Self-awareness is one of the most complex manifestations of human cognition and argued to be a prerequisite for understanding mental states of ‘self’ and ‘others’ (Gallup, 1970; Keenan et al., 1999). Self-awareness exists in different domains, e.g., in the physical domain as the awareness of one’s own body and faces, in the psychological domain as an entity with specific traits and qualities, and in the temporal domain as a continuous being across time (James, 1890). Physical self-awareness is one of the earliest and most basic domains of self-awareness to develop. Among other methods, self-face recognition has been often used as a paradigm to interrogate this domain of self-processing (Amsterdam, 1972; Keenan et al., 2000, 2003; Sugiura et al., 2000; Kircher et al., 2001; Nielsen and Dissanayake, 2004; Uddin et al., 2005; Brédart et al., 2006; Sui et al., 2006; Platek et al., 2008; Devue et al., 2009; Pannese and Hirsch, 2011)and most studies on physical self-representation have focussed on the investigation of the behavioral and neural basis of self-face recognition (Keenan et al., 1999; Tong and Nakayama, 1999; Kircher et al., 2002; Uddin et al., 2005; Platek et al., 2008; Ma and Han, 2010). Comparatively little is known (Kita et al., 2010; Hungr and Hunt, 2012) about gaze behavior during the recognition of a face as belonging to oneself, leading to the question of whether the gaze response pattern for a face recognized as ‘self’ is different from one recognized as ‘other.’ This line of investigation has led to theoretical accounts that question whether self-faces are ‘special’ in any way (Gillihan and Farah, 2005)? The study of eye gaze behavior in self-face recognition allows for better understanding of visual strategies underpinning this fundamental aspect of physical self-awareness. In an age of ‘selfies,’ how we look at our own face assumes an importance beyond the academic domain.

Looking at self-face is associated with greater attention to and faster recall compared to other faces (Tong and Nakayama, 1999; Devue et al., 2009). Identification of self-face requires orientation toward the self from a decentralized position and indicates high salience for self-related stimuli (Heinisch et al., 2011). The self-face is identified faster among other faces even where faces are presented in non-upright conditions (Tong and Nakayama, 1999; though see Devue et al., 2009). Such high salience for self-related stimuli is also evident from their facilitatory effect on spatial priming (Pannese and Hirsch, 2011), interference with cognitive tasks (Brédart et al., 2006), as well as from EEG experiments showing an increased P300 signal (related to attention allocation) to self-name (Gray et al., 2004). Self-specific stimuli have been found to alter the salience of neutral stimuli by association leading to the proposal of the self-attention network (SAN) (Sui and Humphreys, 2013; Porciello et al., 2016). SAN constitute a model where neural networks involved in self-processing interact with attentional networks to determine self-salient behavior. Notably, this individual-specific salience for the self-face is distinct from the salience due to low-level visual features of the presented stimulus.

Traditionally, paradigms investigating self vs. other face representation have presented self or other face photographs (Keenan et al., 1999, 2003; Platek et al., 2008). Few paradigms have used a psychophysics based approach to investigate individual differences in the parameters of the psychometric response function associated with self-other face recognition (Hungr and Hunt, 2012; Chakraborty and Chakrabarti, 2015). In these paradigms, the psychometric response function is calculated based on the participant’s identification of the morphs as ‘self’ or ‘other’ (Chakraborty and Chakrabarti, 2015). However, such paradigms tend not to include simultaneous eye-tracking measures; it is thus not possible to elucidate if the pattern of gaze fixation to self and other faces can predict the mental representation of the self-face. The combination of a psychophysics paradigm with simultaneous eye-tracking enables this study to investigate (a) differences in gaze pattern to face morphs identified as self vs. those identified as other, thus subjective tailoring of the self vs. other face stimuli, and (b) the relationship of psychophysical representation of the self-face with differences in associated gaze behavior.

**FIGURE 1:**
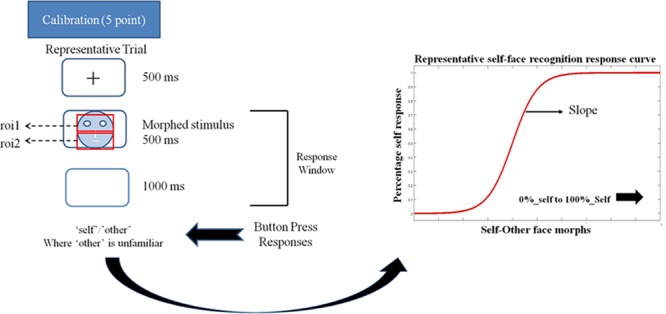
Schematic representation of a trial in the eye-tracking task. Participant’s eye movements and gaze pattern were recorded during the 500 ms stimulus presentation window and the behavioral response was recorded in the 500 + 1000 ms window. Participants pressed the ‘a’ key for identifying a face as ‘self’ and ‘l’ key for identifying a face as ‘other’ in the self left-hand response. These key response contingencies were reversed for the self-right hand response. The schema also shows a representative self-face recognition response curve calculated from the ‘self’/other’ face recognition data.

Using a morphing paradigm, an eye-tracking study reported a stronger gaze cueing effect for self-similar compared to self-dissimilar faces (Hungr and Hunt, 2012). However, visual processing strategies of self and novel faces in previous studies have not been mapped onto the psychometric properties of self-face representation. The current study addresses this gap in knowledge by using a self-other face morphing paradigm with simultaneous eye-tracking to investigate the relationship between self-face representation at the behavioral level (operationalized by the slope of the psychometric function) and gaze patterns for faces identified as ‘self’ versus ‘other.’ In the context of the present study, the ‘self’ and ‘other’ constitute two categories. The steepness of the slope of the psychometric function for the self-face recognition curve (derived from self-other face morphs) provides a measure of the overlap between the two categories. A steeper slope of the self-face recognition curve indicates a lower extent of overlap between self and other categories, i.e., more distinct self-face representation. In other words, it would take small changes in stimulus feature (changes in morph percentages) to shift from the self to other category for an individual with a more distinct self-face representation. Conversely, a shallower slope of the self-face recognition curve indicates a broader spread of category boundary that requires a larger change in stimulus feature to shift from the self to other category (**Figure 1**).

It is predicted that the slope of the self – recognition response curve will be positively associated with greater gaze duration to the eye region for morphed faces identified as ‘self.’ Eyes provide the richest source of information for identification of a face (Laughery et al., 1971; Janik et al., 1978; Emery, 2000; Schyns et al., 2002; Henderson et al., 2005; Itier et al., 2007; Luria and Strauss, 2013). Accordingly, those with a more distinct representation of the self (indexed by a steeper slope in the psychometric response function for self-recognition) are likely to spend more time extracting information from the eyes for faces identified as ‘self.’ This processing strategy is not predicted for the novel other face as there is no previous exposure to the novel face that will direct such gaze behavior.

A secondary aim of the current study is to test individual differences in the self-face processing in relation to autism-related traits. Atypical gaze fixation to social stimuli (Klin et al., 2002; Pelphrey et al., 2002; Dalton et al., 2005) as well as atypical self-processing (Lombardo et al., 2007, 2009, 2010; Uddin et al., 2008) is well-documented in individuals with autism spectrum disorders (ASDs). Accordingly, the current study aimed to test if there was any association between autistic traits and gaze duration to faces in general and if any such association is specific to facial identity (self or other) and/or facial region (upper vs. lower parts of the face). Measurement of autistic traits in the general population can help investigate how autistic symptoms map onto social behavior. Autistic traits are distributed continuously across the population, and individuals with ASD score highly on these measures (Baron-Cohen et al., 2001). Individuals with a clinical diagnosis of ASD typically score at the high end of this continuous distribution of autistic traits. Measuring autistic traits in the general population allows one to measure associations between trait features and experimental manipulations, thus providing an initial foundation for follow-up investigations with the clinically diagnosed tail of the trait distribution (Robinson et al., 2011). In the present study, autistic traits are measured using autism spectrum quotient (AQ) (Baron-Cohen et al., 2001). AQ scores range from 0 to 50, and individuals scoring 32 or higher have >80% chance of having an ASD diagnosis (Baron-Cohen et al., 2001). AQ has been developed based on the behavioral symptoms of ASD and has five subdomains that include social skills, attention switching, and attention to detail, communication, and imagination.

This study investigates the association between autistic traits and gaze duration to upper and lower parts of self and other faces. It is predicted that autistic traits will correlate negatively with gaze duration to the upper portion of the face. This negative association between autistic traits and gaze duration to eye-region is predicted to be stronger for faces identified as ‘other’ compared to those identified as ‘self.’ One of the theoretical explanations for reduced gaze to the eye region in ASD suggests it to be a negative and stressful reaction to eye-contact in individuals with ASD (Hutt and Ounsted, 1966; Kliemann et al., 2010), a reaction that may not hold true for self-faces.

An alternative theoretical account from, the social motivation theory of ASD posits that reduced fixation to eye region is driven by reduced reward values associated with social stimuli in ASD (Chevallier et al., 2012) with an increased preference for geometrical images compared with social images observed in children with ASD (Pierce et al., 2011, 2016). If this theory holds true it can be expected that the association between higher autistic traits with reduced gaze to eye regions to be less severe for faces identified as ‘self.’ This is predicted because self-face can be argued to be of higher reward value (Devue et al., 2009).

## Materials and Methods

### Participant Details

Thirty-three healthy adults (two males; mean ± SD age = 20.67 ± 3.69 years) were drawn from in and around the University of Reading campus and received either a small compensation or credit points for their participation. All participants were right-handed and had normal or corrected to normal vision. None of the participants had a current clinical diagnosis of neurological or psychiatric disorder and did not self-report any mental health problems. Ethical approval for the study was obtained from the Department of Psychology Research Ethics Committee of the University of Reading and all methods were carried out in accordance with these guidelines regarding all relevant aspects, such as recruitment, compensation, and debriefing of participants, as well as the nature of the experiments and other collected information. All participants provided written informed consent in accordance with the Declaration of Helsinki.

### Stimuli

Stimuli were individually tailored for each participant. Each participant was photographed (Canon PowerShot SX700 HS digital camera) looking directly at the camera and holding a neutral expression. Participants were seated at a distance of 100 cm, under constant artificial lighting and with a white background. One novel ‘other’ identity for each gender and from the same ethnicity and age range was also photographed under similar condition.

Following this, each participant’s photograph was converted to grayscale and external features (hairline, jaw line, and ears) were removed. This photograph was then mounted on an oval frame and cropped to a dimension of 350 × 500 pixels using GIMP (2013). A set of stimuli was created for each participant’s face, by morphing self-face with an ‘novel faces’ using Sqirlz Morph (Xiberpix, Solihull, United Kingdom). The following step sizes were used to create the morphing continuum from 100 to 0% of participant’s face (100, 90, 80, 70, 65, 60, 55, 50, 45, 40, 35, 30, 20, 10, 0). Since the previous data showed that individual differences in self-other face category boundary lie within the morph range of 60 and 30 morph percentages for the self-face recognition task (Chakraborty and Chakrabarti, 2015), the morph percentages were at 10% step sizes at the two ends of the continuum, and 5% step sizes between 70% and 30%.

### Apparatus

Calibration and task presentation were controlled using E-prime 2.2 (Psychological Software Tools, Pittsburgh, PA, United States) presented with TobiiStudio on a Tobii T60 eye tracker monitor (operating at 60 Hz) with a resolution of 1280 × 1024 pixels. Participants sat in a chair 50 cm from the monitor. They used a keyboard for their responses to the task.

### Eye-Tracking Measurements

Before commencing the task, participants underwent a five-point calibration procedure implemented on Tobii Studio.

Next, participants completed a self-face recognition task. Each trial started with fixation cross (500 ms) followed by the stimulus image (500 ms) and then a blank screen (1000 ms) (see **Figure 1**). Participants were instructed to classify a presented face as either ‘self’ or ‘other’ using the keyboard (using the key ‘a’ with the left hand or ‘l’ with the right hand) within the 1500 ms response window (500 + 1000 ms). Any keyboard response in the 1500 ms window was recorded. There were two runs for each task, and keys associated with ‘self’ and ‘other’ responses were switched between runs. Each run consisted of 15 distinct morphs presented 10 times each, resulting in 150 trials per run. The order of runs was counterbalanced across participants.

Faster recognition of self-compared to other faces has been associated with right hemispheric dominance, i.e., people are slightly quicker to recognize self-faces when people respond with their left hand (Keenan et al., 1999). We collected responses from both hands to ensure that the effects of interest were not influenced by similar potential hemispheric dominance effects.

All participants completed the AQ questionnaire online following the completion of the task. None of the tested participants had AQ of 32 or higher which is considered to be the cut-off threshold for a clinical diagnosis.

### Data Analysis

Statistical tests were conducted and plots generated using SPSS 21 (IBM SPSS Statistics version 21) and R using ggplot2 package (Wickham, 2009).

Slope calculation for self-other recognition: ‘Self’ and ‘other’ responses for both runs were combined for each morph level to generate percentage response curves for self-face recognition response for each participant. The slope of self-recognition for each participant was calculated using a logistic psychometric function fitted for maximum likelihood estimation for Weibull distribution. Depending on the stimulus-related information change across the different morph levels required by an individual participant to shift from the self to other category, the psychometric function gives a steep or shallow slope (see **Figure 1**). The steepness of this slope is interpreted as an extent of overlap between the self-face and other face representation. A steeper slope indicates a reduced overlap between the self and other representation. In other words, a steeper slope represents a more distinct self-representation.

AQ score for each participant was calculated using the formula as suggested by the authors (Baron-Cohen et al., 2001).

#### Gaze Duration Analysis

Two regions of interest (ROIs) were pre-positioned over each morphed face for each individual participant. The first region of interest (*UPPER ROI*) covered the upper portion of the face including the eyes. The second region of interest (*LOWER ROI*) covered the lower portion of the face including the mouth. Both ROIs were of the same size (**Figure 1**). Gaze position, as well as the ROI where gaze was on, was recorded using E-prime for each time stamp. Gaze position was determined by averaging the locations of both eyes. In the absence of one eye position during the time stamp, the eye position for the single recorded eye was used. The data were processed using MATLAB (MathWorks, 2017). The following criteria were used to identify fixations to be included in the analysis:

(1) Three successive time stamps within 35 pixels of the original time stamp. Each time stamp is approximately 16.6 ms long, hence for a fixation to be included the eye position needed to be within the region of interest for a minimum of 50 ms.
(2) If gaze was outside the range for one time stamp (possibly due to blinking) or was not recorded but the following time stamp was inside the range, the fixation was considered legitimate.

Following the gaze position analysis, the average gaze duration to ***UPPER ROI*** was calculated for each participant for all trials that the participant identified as ‘self-face’ (*Average_upper_duration_self*) and ‘other-face’ (*Average_upper_duration_other*). Similarly, the average gaze duration to ***LOWER ROI*** was calculated for each participant for all trial identified as ‘self-face’ (*Average_lower_duration_self*) and ‘other-face’ (*Average_lower_duration_other*).

In each participant, total gaze duration for self-face was calculated by adding the *Average_upper_duration_self* and *Average_lower_duration_self*. Total gaze duration for other-face was calculated by adding the *Average_upper_duration_other* and *Average_lower_duration_other*.

Next, the proportion of gaze duration to *UPPER ROI* (*Upper_proportion_self*) was calculated for each participant for all trials identified as ‘self-face’ by dividing Average_upper_duration_self by the sum of Average_upper_duration_self and Average_lower_duration_self (see **Box 1**). A similar calculation was done for faces identified as ‘other.’ The denominators in both instances were chosen to control for individual differences in total looking time to the different ROIs.

**Box 1:**
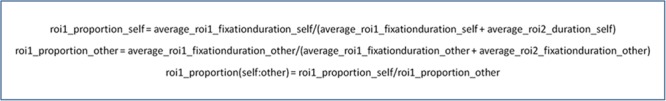
Formulae used to calculate metrics for gaze duration to *UPPER ROI* and *LOWER ROI* controlling for total gaze duration to both the ROI-s for all faces identified as ‘self’ and ‘other’ for each participant.

### Data Analysis

#### Normality Checks and Exclusions

The distribution of all variables was tested before analysis, using Shapiro–Wilk test of normality. Parametric and non-parametric tests of statistical inference were used accordingly (see **Table 1**). Influence measures (Cook’s D and leverage) were calculated for each correlation and data points exceeding a cut-off of 4/N were excluded from correlation analysis. Due to the strong directionality of the predictions, one-tailed statistics are used except for the exploratory analysis between AQ and gaze behavior.

**Table 1:** Mean and SD for the computed variables.

Variables	Mean (*SD*)	Shapiro–Wilk statistics
Upper_proportion_self	0.84 (0.19)	*W* = 0.790, *p* < 0.001^∗^
Upper_proportion_other	0.86 (0.178)	*W* = 0.75, *p* < 0.001^∗^
Slope of self-face recognition	14.9 (7.2)	*W* = 0.89, *p* = 0.002^∗^
Threshold of self-face recognition	43.03 (5.56)	*W* = 0.73, *p* < 0.001^∗^
Average_upper_duration_self (ms)	282.47 (93.3)	*W* = 0.960, *p* = 0.3
Average_upper_duration_other (ms)	286.88 (91.2)	*W* = 0.936, *p* = 0.05
Average_lower_duration_self (ms)	47.88 (52.8)	*W* = 0.823, *p* < 0.001^∗^
Average_lower_duration_other (ms)	40.28 (43.3)	*W* = 0.83, *p* < 0.001^∗^
AQ	16.4 (6.34)	*W* = 0.83, *p* = 0.83

*Multiple variables violated the assumption of normality. ^∗^Depicts a significant deviation from the assumption of normality.*

#### Main Effects Analysis

To investigate the difference in relative gaze duration to the different parts of the face (upper/lower), for faces identified as ‘self’ and ‘other’ a related sample Wilcoxon signed-rank test was computed.

#### Individual Differences Analysis

To investigate individual differences in the association between the slope of the self-recognition curve and gaze duration, Kendall rank correlations were computed between the slope of the self-face recognition response curve and (a) the Upper_proportion_self and (b) Upper_proportion_other. To investigate individual differences in the association between AQ scores and eye gaze duration, Kendall rank correlations were computed between AQ and (a) Upper_proportion_self and (b) Upper_proportion_other.

## Results

### Main Effects

**FIGURE 2:**
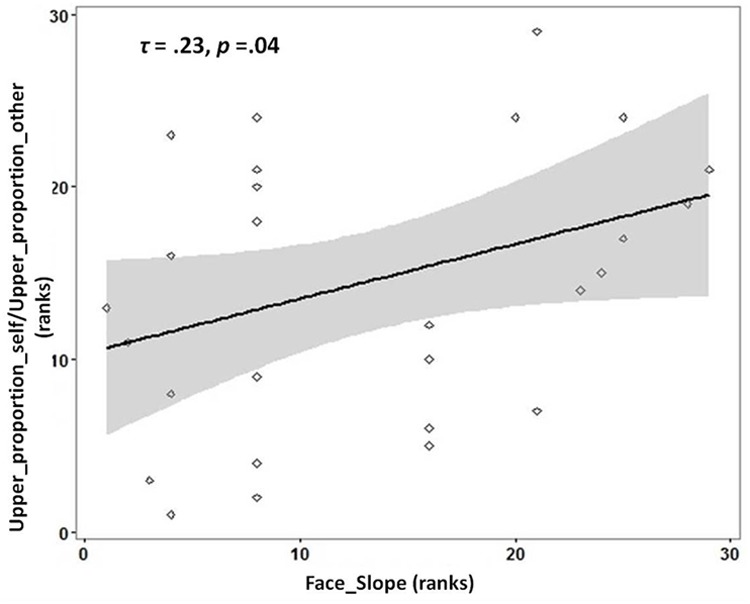
Rank scatterplot representing the positive association between the slope for self-face recognition (x-axis) with the proportion of gaze duration (y-axis) to *UPPER ROI* [ratio_proportion (self:other)] for faces identified as ‘self’ compared to faces identified as ‘other.’ The shaded portion represents the 95% confidence region of the slope of the regression line.

There was a significantly greater proportion of gaze duration to *Lower ROI* for morphed faces identified as ‘self’ compared to morphed faces identified as ‘other’ (Wilcoxon Signed-Rank test: *Z* = -2.385, Asymp.Sig = 0.02, effect size *r* = 0.42). Average_upper_duration_self (Mean = 282.47; *SD* = 93.28) and Average_upper_duration_other (Mean = 286.88, *SD* = 91.19) did not differ significantly from each other [*t*(32) = -1.363, *p* = 0.182]. However, Average_lower_duration_self was significantly different from Average_lower_duration_other [*t*(32) = 2.940, *p* = 0.006]. To investigate if individuals with more distinct self-face representation would gaze longer at the upper parts of faces identified as ‘self’ in proportion to faces identified as ‘other,’ the ratio of gaze duration for Upper_proportion_self to Upper_proportion_other was chosen as the dependent variable. This ratio was chosen as it provides, for each participant, a measure of whether the relative gaze duration to the upper parts of faces is higher for faces identified as self vs. those identified as ‘other.’ In line with the prediction, a significant positive correlation was observed between the slope of self-face recognition with the ratio of Upper_proportion_self to Upper_proportion_other [Kendall’s tau: *τ*(29) = 0.23, *p* = 0.04; see **Figure 2**].

No effect of the responding hand (left/right) was noted on the gaze duration to faces identified as ‘self’ (*t* = -1.79, *p* > 0.05) or ‘other’ (*t* = 0.6, *p* > 0.05). Covarying out gender in the analyses reported above did not show any significant change.

### Individual Differences

No significant association was noted between autistic traits and proportion of relative gaze duration to *UPPER ROI* for faces identified as ‘self’ [Upper_proportion_self; Kendall’s tau: *τ*(33) = -0.008, *p* = 0.48] or ‘other’ [Upper_proportion_other; Kendall’s tau: *τ*(33) = 0.02, *p* = 0.45].

**FIGURE 3:**
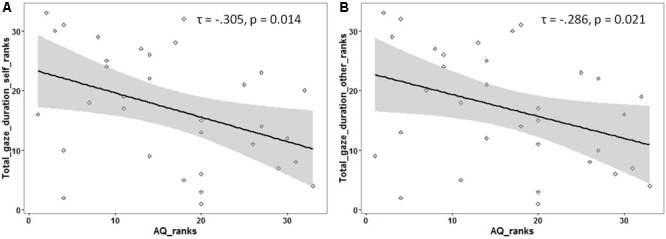
Rank scatterplots representing the negative association between the AQ scores with the total gaze duration for faces identified as **(A)** ‘self’ and for faces identified as **(B)** ‘other.’ The shaded portion represents the 95% confidence region of the slope of the regression line.

Following previous findings of reduced overall looking time to social stimuli (like faces) in individuals with ASD, an exploratory analysis was carried out to investigate if the total looking time to faces (adding the gaze duration for both ROIs) was associated with autistic traits. The total gaze duration (for each participant) was calculated for faces identified as ‘self’ and as ‘other.’ A significant negative correlation was observed between autistic traits and total looking time for faces identified as ‘self’ [Kendall’s tau: *τ*(33) = -0.305, *p* = 0.01] as well as faces identified as ‘other’ [Kendall’s tau: *τ*(33) = -0.286, *p* = 0.02; see **Figure 3**] (see Supplementary Material for raw data plots).

In line with previous results (Chakraborty and Chakrabarti, 2015), no significant association was observed between the self-face recognition slope and autistic traits [Kendall’s tau = *τ*(33) = -0.120, *p* = 0.2].

## Discussion

This study tested differences in gaze pattern for faces identified as ‘self’ and ‘other’ from a series of self-other face morphs. The study also investigated the association of these gaze patterns with the behavioral representation of self-faces and autistic traits. We found a significant difference in the proportion of gaze duration to upper vs. lower regions of a face between faces that were identified as ‘self’ and those identified as ‘other.’ We also found that individuals with a more distinct self-face representation looked longer at the upper region of faces identified as self vs. those identified as other. Contrary to our predictions, no significant association was observed between autistic traits and the proportion of gaze duration to upper parts of morphed faces for faces identified as ‘self’ or for faces identified as ‘other.’ However, a negative association between autistic traits and total gaze duration to both faces identified as ‘self’ and as ‘other’ was noted. The results are discussed in details in the following paragraphs.

### Increased Facial Feature Sampling for Faces Labeled As ‘Self’ Compared to Those Labeled As ‘Other’

Self-face has high relational salience (Brédart et al., 2006) to the individual and may possess high subjective reward value (Devue et al., 2009). However, the visual processing strategies employed in recognizing the highly salient and familiar self-face is relatively unknown.

Greater proportion of gaze was allocated to the lower parts of the face for faces labeled as ‘self’ compared to those labeled as ‘other.’ To further understand the differences seen in the proportion data, the average duration to upper and lower ROI for self and other faces was compared. Faces identified as ‘self’ were found to have longer average gaze duration to lower parts of the face compared to those identified as ‘other.’ These results are consistent with previous studies that have reported increased feature sampling for familiar faces across different regions of the face (Van Belle et al., 2010). Gaze allocation strategies for identification of familiar and novel faces are different from each other and known to be task-dependent. Furthermore, a simultaneous eye-tracking and fNIRS study of self-face and familiar face recognition did not find any difference in gaze fixation patterns between self-face and familiar faces (Kita et al., 2010). Together, the current results and the previous studies point to the close similarities in gaze allocation strategies to self and familiar faces, which are distinct from those for novel faces.

Notably, however, the average gaze duration to the upper region of the face was not significantly different between the two identities. This is in line with the well-established findings that gaze duration is longer for eye regions compared to other regions of the face, irrespective of identity (Laughery et al., 1971; Janik et al., 1978; Emery, 2000; Schyns et al., 2002; Henderson et al., 2005; Itier et al., 2007; Luria and Strauss, 2013). Since gaze duration to the upper ROI was comparable for both self and other faces, we believe the longer gaze duration to lower parts of the face for faces identified as self-face could be attributed to the greater exploration of facial features of self-face. Self-face has been found to sustain attention (Devue et al., 2009). We conclude that even if information extracted from the eye-region is sufficient for an individual to identify a face as self-face, increased feature sampling from different regions indicates possible rewarding nature of self-face with its ability to sustain attention. However, it is not known how much of the observed pattern of results are driven by the nature of the stimuli used for self and other faces in the current study (static faces with closed mouths). It is possible to speculate that gaze allocation strategies will be different for dynamic faces, particularly speaking faces, which can lead to increased gaze on mouth region for ‘other’ faces because of its role in verbal communication. Future studies should explore this possibility.

### More Distinct Self-Face Representation Associated with Greater Sampling of Upper Region of Faces Labeled As ‘Self’

The slope of the psychometric function for self-recognition was positively associated with the ratio of gaze proportion to the upper region for faces identified as ‘self’ to those identified as ‘other.’ This finding suggests that individuals with a more distinct self-face representation spent a greater proportion of time looking at the upper part of faces (including the eye region) for faces labeled as ‘self.’ Due to the correlational nature of the study, it is not possible to infer directionality of this association. This observation raises questions about the stability of self-face representation, and the impact of task manipulations on it. If self-face representation is influenced by task conditions, a future experiment could explicitly ask participants to look at the upper vs. lower regions of the face, or present the face eccentrically, to test if and how these manipulations alter the slope of the self-face representation.

The current study did not compare self-face with familiar other faces. Depending on the exposure level, a familiar face may also be of high salience and well-represented mentally. Follow-up research should test if distinct behavioral representations of familiar other faces are associated with increased gaze duration to upper parts of these faces.

### Gaze Duration to Faces Is Reduced with Higher Autistic Traits, Irrespective of Identity

Reduced gaze duration to both self and other faces was noted in individuals with high autistic traits. The negative correlation between autistic traits and total gaze duration to faces replicates several previous reports where individuals with ASD have been shown to demonstrate reduced gaze duration toward faces (Pelphrey et al., 2002; Dalton et al., 2005). However, no significant difference in this association was noted for self vs. other faces. The lack of an identity effect on gaze allocation to faces echoes previous results in children and adults with ASD (Sterling et al., 2008). This observation suggests that (a) individuals are performing at ceiling due to the relative ease of the task, thus masking any potential difference between the processing of self vs. other faces, or (b) the differences in gaze processing strategies between self and other faces are orthogonal to the dimension of autistic traits. Despite not showing a main effect of facial identity, one of these studies had observed a negative association between socio-communicative abilities and gaze patterns to self and novel faces (Gillespie-Smith et al., 2014). The current study is consistent with this earlier report.

As one of the first studies to combine eye movement recording with a psychophysics paradigm to measure self-face recognition, this study has some limitations that offer useful directions for future research. We discuss five such directions below. First, this paradigm did not include familiar other faces as a stimulus category. To directly compare the self-face related results from this study with previous eye-tracking studies investigating familiar face recognition, future experiments should include both these conditions in the same task. Second, the sample for the current study is strongly biased in favor of females and was therefore not equipped to explore gender differences which should be explored in future studies with more balanced samples. Third, this study presented the faces at the center of the screen instead of an eccentrically localized position on the screen which could have resulted in initial fixation to be localized on the center of gravity of the presented face. Future studies should consider the eccentric presentation of the faces to address this potential confound. Fourth, this task was not optimized for measuring response times. Participants were not given any instruction on how quickly to respond, which might have led to different strategies employed by different participants. Finally, the scope the current study is also limited in terms of the trait measures that it investigates. While autistic traits are of interest to self-face processing, they are by no means the only dimensions that can be theoretically linked to potential differences in these processes. Future research should examine other traits that could relate to individual differences in self-face representation and associated gaze behavior to self-faces. For example, do individuals who exhibit a more distinct self-face representation exhibit preoccupation with their body image?

## Conclusion

This study shows that the visual processing of faces identified as ‘self’ is different from those identified as novel other. These differences in visual processing are associated with individual differences in self-face representation. Individuals with a ‘more distinct’ self-face representation spent a greater proportion of time looking at the upper regions of faces identified as self, compared to those identified as other. The results from this study support the idea that self-faces might be processed similarly to other familiar faces, and self-specificity effects may come into play at higher order relay regions in the brain (Kita et al., 2010).

**FIGURE 4:**
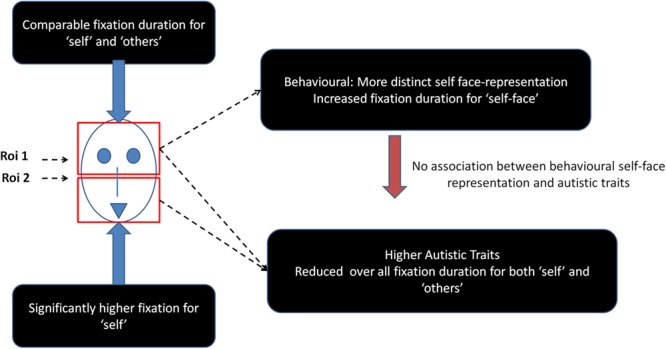
Schematic representation of the main findings from the study.

This study also shows that higher autistic traits do not specifically influence gaze responses to self-face but reduce looking time to faces (self and other) in general (see **Figure 4** for a summary of results). Consistent with a previous report (Chakraborty and Chakrabarti, 2015) in a similar neurotypical population, this study found that psychometric properties of physical self-representation is uninfluenced by autistic traits. This observation lends support to the domain-specific influence of autistic traits on self-representations (Williams, 2010). However, physical self-representation needs to be formally tested in a clinically diagnosed ASD population using similar approaches to test the generalisability of the current results to the extreme high end of the spectrum of autistic traits.

## Author Contributions

Both authors developed the study concept. The study design, data collection, analysis, interpretation, and draft of the manuscript were performed by AC under the supervision and critical revisions of BC. Both authors approved the final version of the manuscript for submission.

## Conflict of Interest Statement

The authors declare that the research was conducted in the absence of any commercial or financial relationships that could be construed as a potential conflict of interest.
