# Supplementary material for: Looking at My Own Face: Visual Processing Strategies in Self–Other Face Recognition
Source: Front Psychol. 2018 Feb 13;9:121. doi: 10.3389/fpsyg.2018.00121 (PMC5816906; doi:10.3389/fpsyg.2018.00121)
Supplement: Supplementary file 1 [file Image_1.pdf]

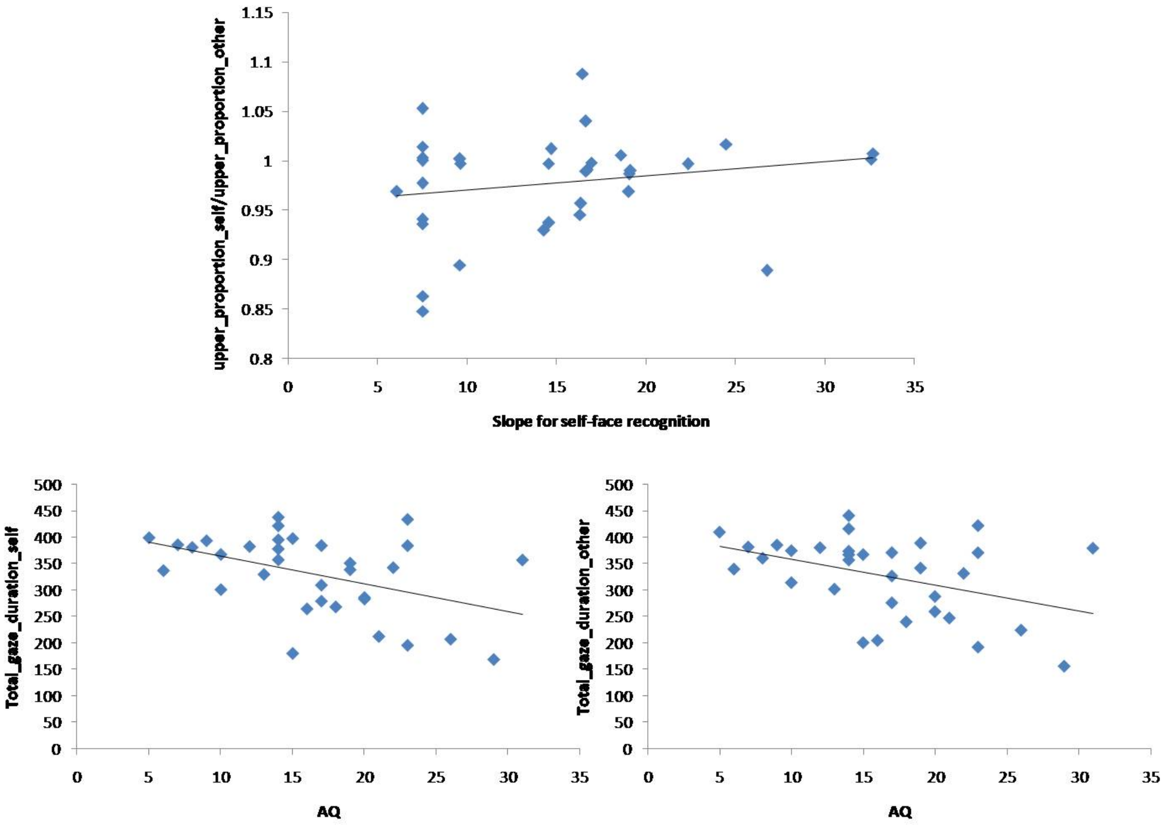

Supplementary Figure S1:

Top panel : Scatterplot of the raw data representing the association between the slope for self-face recognition (x-axis) with the proportion of gaze to UPPER ROI for faces identified as 'self' compared to faces identified as 'other.'

Bottom left : Scatterplot of the raw data representing the association between AQ with the total gaze duration for faces identified as 'self'.

Bottom right: Scatterplot of raw data representing the association between AQ with the total gaze duration for faces identified as 'other'.
